# Contrasting Expression of Keratins in Mouse and Human Embryonic Stem Cells

**DOI:** 10.1371/journal.pone.0003451

**Published:** 2008-10-20

**Authors:** Jochen Maurer, Brandon Nelson, Grace Ceceña, Ruchi Bajpai, Mark Mercola, Alexey Terskikh, Robert G. Oshima

**Affiliations:** Burnham Institute for Medical Research, La Jolla, California, United States of America; Baylor College of Medicine, United States of America

## Abstract

RNA expression data reveals that human embryonic stem (hES) cells differ from mouse ES (mES) cells in the expression of RNAs for keratin intermediate filament proteins. These differences were confirmed at the cellular and protein level and may reflect a fundamental difference in the epithelial nature of embryonic stem cells derived from mouse and human blastocysts. Mouse ES cells express very low levels of the simple epithelial keratins K8, K18 and K19. By contrast hES cells express moderate levels of the RNAs for these intermediate filament proteins as do mouse stem cells derived from the mouse epiblast. Expression of K8 and K18 RNAs are correlated with increased c-Jun RNA expression in both mouse and human ES cell cultures. However, decreasing K8 and K18 expression associated with differentiation to neuronal progenitor cells is correlated with increasing expression of the Snai2 (Slug) transcriptional repression and not decreased Jun expression. Increasing K7 expression is correlated with increased CDX2 and decreased Oct4 RNA expression associated with the formation of trophoblast derivatives by hES cells. Our study supports the view that hES cells are more similar to mouse epiblast cells than mouse ES cells and is consistent with the epithelial nature of hES cells. Keratin intermediate filament expression in hES cells may modulate sensitivity to death receptor mediated apoptosis and stress.

## Introduction

Keratin intermediate filament proteins, keratin 8 (K8, Krt8, EndoA) and keratin 18 (K18, Krt18, EndoB) were first identified in liver and as markers of mouse embryonal carcinoma (EC) and embryonic stem (ES) cell differentiation [1]–[3]. Investigation of early mouse embryos confirmed that the differentiation of the inner cell mass of mouse blastocysts to trophoblast derivatives and extra-embryonic endoderm parallels the induction and accumulation of K8 and K18 [4]–[6]. The mouse inner cell mass initially expresses low amounts of K8/K18 intermediate filaments but then represses expression [7], [8]. Many studies have confirmed the low levels of both protein and RNA for K8 and K18 in mouse ES cells in their undifferentiated state. The isolation of human ES cells that express transcription factors associated with pluripotentiality in mouse ES cells (Oct4, Sox2, Nanog) led to the expectation that epithelial keratins K8 and K18 might be expressed at similar low levels as mES. [9]. Recently a new pluripotent stem cell, the epiblast stem cell (EpiSC), derived from post-implantation mouse embryos was isolated and characterized by two different laboratories [10], [11]. While ES cells and EpiSC cells share characteristics of pluripotency such as the expression of *Oct4*, *Sox2* and *Nanog*, the gene expression profile of EpiSC includes markers of the embryonic epiblast and resembles human ES cells more than mouse ES cells. Our analysis of the published RNA expression profiles of hES, mES and EpiSC reveals that K8 and K18 RNAs are greatly elevated in EpiSC.

Here we show that expression of K8, K18 and K19 is characteristic of hES cell lines in the undifferentiated state and contrasts with mES cells but is similar to EpiSC. The differentiation of hES to neuronal progenitors results in decreased expression of these keratins and elevated expression of neuronal markers while the spontaneous differentiation of hES cells to presumptive extraembryonic endodermal derivatives results in increased accumulation of K18. Expression of K8, K18 and K19 are characteristic of the epithelial nature of undifferentiated hES cells and contrasts with mouse ES cells.

## Results

### Differential expression of keratin RNA in human and mouse ES cells

**Figure 1:**
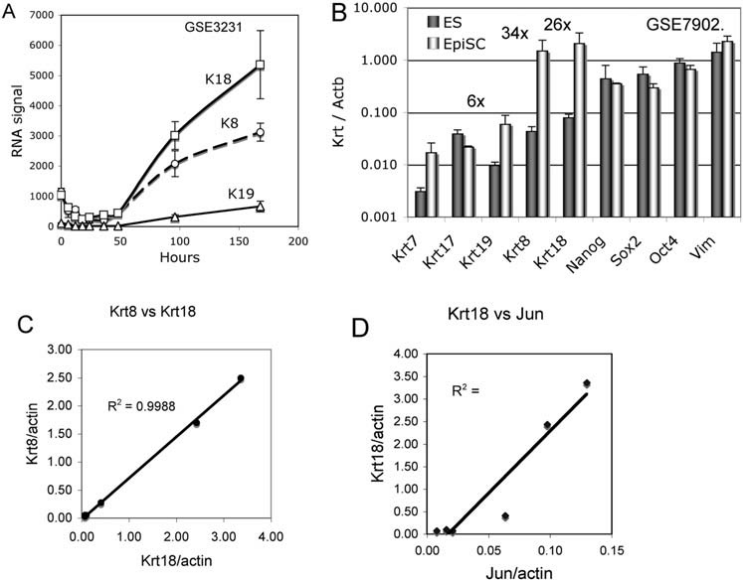
Keratin RNA expression in mouse ES cells and EpiSCs. A, time course of K8, K18 and K19 RNA induction in mouse ES cells during embryoid body differentiation plotted from supplementary data of a published report [33]. Data set is identified by the GSE number of the GEO data base. B, contrasting expression of K8, K18 and K19 in mouse ES and epiblast stem cells (EpiSC). RNA expression data [10] for the indicated genes was compared by normalizing the values of the two cell types to the corresponding signals for beta-actin RNA. Note the elevated expression of K8 (34×) and K18 (26×) in EpiSC while RNAs for Nanog, Sox2 and Oct4 are similar in the two cell types. C, coordinate variation of K8 and K18 RNAs in individual array values of ES and EpiSC samples from GSE7902. D, strong correlation between K18 and Jun RNA levels. Jun is a component of the AP-1 transcription factor activity previously identified as important in the induction of K18 RNA in differentiation mouse ES cells.

The RNAs for simple epithelial keratins K8, K19 and K19 are expressed at low levels in undifferentiated mES and embryonal carcinoma (EC) cells [2], [3]. Figure 1A shows a typical time course of induction of K8, K18 and K19 RNAs as measured by cDNA array analysis, during the differentiation of mES cells as embryoid bodies. The baseline expression of these genes in undifferentiated ES cultures varies with the extent of contamination from spontaneously differentiated cells. In contrast to mES cells, pluripotent epiblast stem (EpiSC) cells have greatly increased levels of K8 (34 fold), K18 (26 fold) and to a less extent K19 (6 fold) while the pluripotency factors Nanog, Sox2 and Oct4 (Pou5f1) vary little between the two different cell type (Figure 1B). The expression of K8 and K18 RNAs appear coordinately regulated (Figure 1C). Furthermore the increased expression of K8 and K18 RNA in EpiSC is directly correlated with increased expression of Jun (Figure 1D), a component of AP1 transcription factor activity and previously identified as a key regulatory of K18 gene regulation [12], [13], [14], [15]. We suggest that epithelial keratin gene expression is consistent with the definitive polarized epithelial nature of the mouse epiblast.

**Figure 2:**
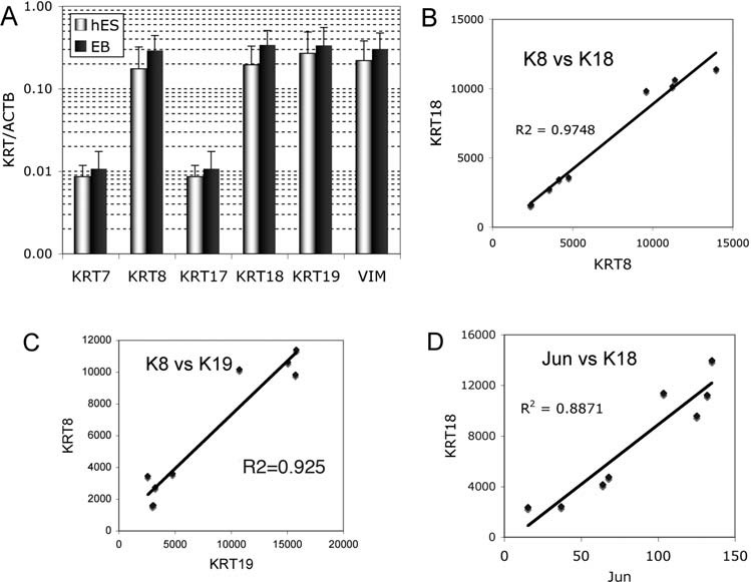
Keratin RNA expression in hES cells. A, published results of hES cells and embryoid body cultures [17]. Data was downloaded from http://www.stemcellcommunity.org/. Averages and standard deviations of normalized data of six undifferentiated cell cultures (WA09, BG01, BG02, BG03) and three embryoid body (EB) cultures (BG01, BG02, BG03). Keratin RNA expression in EBs was less than 2× greater than stem cultures. B, coordinate levels of K8 and K18 RNAs. Values for single arrays of different conditions are compared. C, coordinate values of K8 and K19 RNAs. D, correlation of K18 RNA values with Jun RNA, a key transcriptional regulator of the K18 gene.

In contrast to mES cells, publicly available data show that hES cells and human embryonal carcinoma cells express significant levels of K8, K18 and K19 RNAs [9], [16]–[18]. One example is shown in Figure 2A in which keratin RNA expression of undifferentiated hES cells is compared to cells grown as embryoid bodies. K8 and K18 RNAs are easily detected in hES lines and is modestly increased during embryoid body differentiation. These data are supported by a meta analysis of 38 different array experiments. [19] (http://amazonia.montp.inserm.fr/). While K8 and K18 are expressed in hES cells, differentiation of the cells commonly results in higher expression and thus resulted in the identification of K8, K18 and K19 as under expressed in hES cells compared to differentiated cell types.

Examination of the data of individual array experiments revealed that K8 and K18 levels are very tightly correlated (Figure 2B). K19 RNA is also correlated with K8 levels (Figure 2C). In addition, like mES and mEpiSC cells, K18 RNA expression is associated with increased Jun RNA expression (Figure 2D). One quantitative difference between hES and mEpiSC is the relative expression level of K19, which is as strong as K18 in hES cells. While comparison of hES and mEpiSC and mES cells depends on similar sensitivities of measuring K19 and K19 RNAs, western blot analysis of the proteins support the view that hES cells may have greater relative contribution from K19 than mES or mEpiSC cells.

### K19 is highly expressed in hES cells

**Figure 3:**
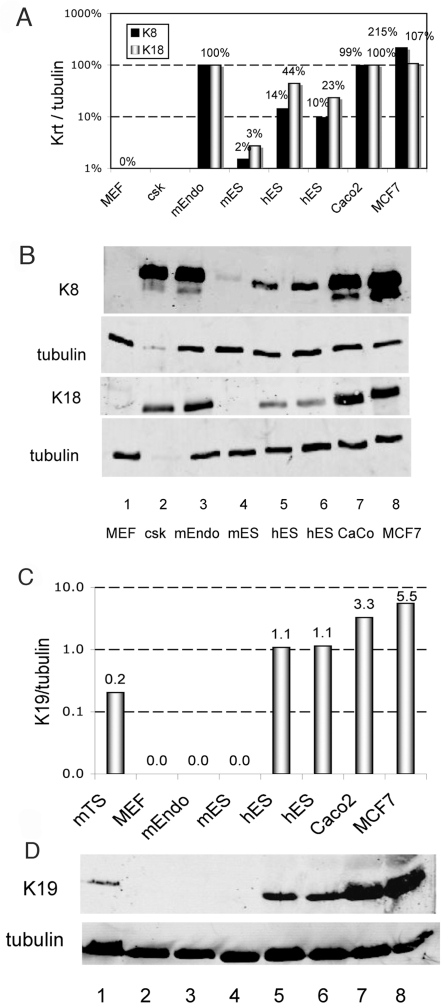
Keratin protein expression in hES cells. Western blots for K8 and K18 were performed with antibodies that recognize both the mouse and human forms of K8 (TROMA1) [6] and K18 (1589) [40]. Antibody reactions were detected and quantitated with Infrared dye labeled secondary antibodies and the LI-COR Odyssey imaging system. A, keratin protein signals were divided by the corresponding tubulin signals. K8 and K18 normalized values were set to 100% for mEndo cells. B, digital image of secondary antibody reaction; MEF (1), mouse embryonic fibroblast; csk (2), non-ionic detergent insoluble fraction of mEndo cells; mEndo (3), the HR9 mouse extraembryonic endodermal cells (4) [43]; Human ES cell lines (5,6); CaCo (7), human colon carcinoma cell line; MCF7 (8), human breast cancer cell line. The upper two panels are from one filter and the bottom two panels are from a second duplicate filter. C, ratio of K19 signal to tubulin signal after background subtraction. D, K19 protein expression in hES cells. Western blot reaction with the L2PK mouse monoclonal antibody to K19 [41], [42]. Tubulin was detected after stripping the K19 antibody. Lanes 1–8 are defined as above.

To confirm expression of K8 and K18 in hES cells, we performed western blot analysis with antibodies that recognized both mouse and human forms of K8, K18 and K19. Typical results for K8 and K18 are shown in Figure 3. Human ES cells express moderate levels of K8 and K18 proteins (Figure 3B, lanes 5,6) compared to mouse parietal endodermal cells (Figure 3A, B, lane 3). Mouse ES cells express very low levels of the two keratin proteins (Figure 3A, B, lane 4) as expected from the low RNA levels. Human ES cells also express significant levels of K19 (Figure 3C, 3D) while neither mES nor a mouse parietal endodermal cell line had detectable levels of K19 protein. K19 expression is detectable in trophoblast stem cells and increases upon differentiation in culture (data not shown). These results were confirmed with several additional antibodies that were species specific for either mouse or human keratins. While hES cells express significant levels of K8, K18 and K19, human Caco2 colon carcinoma cells and MCF7 breast cancer cells (Figure 3B, 3D, lanes 7 and 8) express 3–20 fold higher levels of these proteins. These results indicate that hES cells differ from mES cells with regard to expression of K8, K18 and K19 RNA and protein. With mES cells, this work confirms previous studies of mES and mEC cells [2], [12], [13], [20].

### Human ES cell keratin filaments

**Figure 4:**
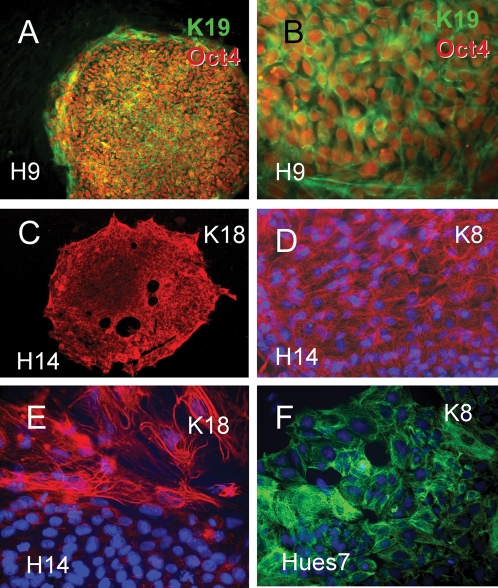
Immunofluorescent detection of K8, K18 nd K19 in hES cells. A, low magnification of H9 hES cells surrounded by MEF feeder layer double stained for Oct4 (red) and K19 (green). Feeders do not react with either antibody. B, high magnification of colony shown in A, showing single Oct4 positive ES cells contain K19 positive filaments. C, low magnification of H14 hES cell colony grown on Matrigel without feeders and stained for K18. D, high magnification of H14 hES cells showing K8 network. Nuclei are stained with DAPI (blue). E, spontaneous differentiated cells at edge of H14 hES colony have increased size and K18 expression. F, example of K8 staining of Hues7 hES cell line.

Both mouse and human ES cultures commonly develop variable numbers of spontaneously differentiated, fibroblast-like and extraembryonic endodermal cells. Immunofluorescent staining of human ES cells was performed to confirm that keratin expression was due to ES stem cells and not the differentiated progeny. Figure 4 shows typical results of K8, K18 and K19 localization in hES cells. At low magnification, a colony of hES cells grown with mouse fibroblast feeder cells is relatively uniformly reactive with antibodies to both Oct4 and K19 (Figure 4A,B). The mouse feeder cells do not react with either Oct4 or K19 antibodies (Figure 4A). Human ES cells express both K18 (Figure 4C) and K19 (Figure 4A,C) as type I keratin filament proteins. K8 is the primary complementary type II keratin expressed in these epithelial cells (Figure 4D). Similar results were obtained for the H9, H14 and Hues7 human ES cell lines (Figure 4F) and in the presence or absence of feeder layers.

### Keratin changes associated with the differentiation of hES cells

**Figure 5:**
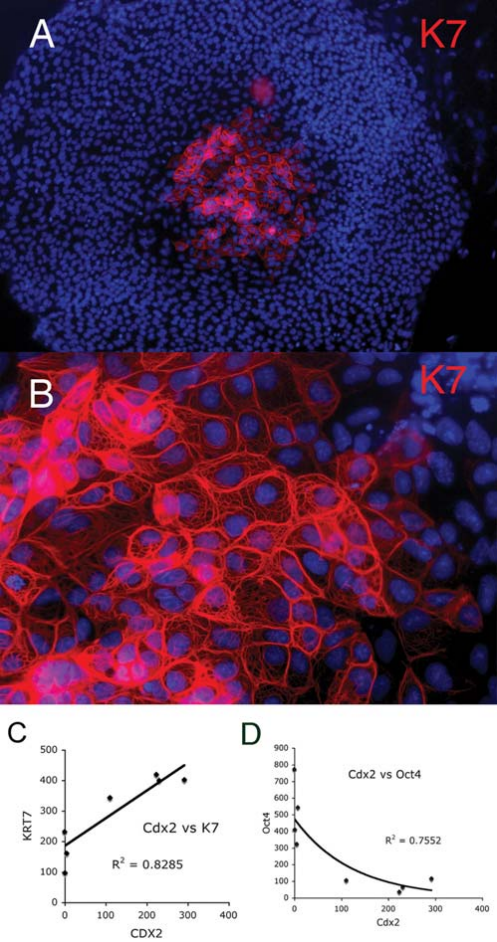
Presumptive trophoblast differentiation of H9, hES cells. A, K7 positive colony of cells forming within a H14 hES cell colony. Nuclei are stained with DAPI (blue). B, higher magnification showing extended cytoskeletal pattern of K7 with increased densities at intercellular borders. C, K7 RNA expression is correlated with Cdx2 RNA expression in hES cell cultures [17]. Cdx2 is a master regulator of trophoblast differentiation. D, Cdx2 RNA is inversely correlated with Oct4 RNA expression in differentiating cultures of hES cells.

Spontaneous differentiation of both human and mouse ES cells occurs commonly, even under conditions promoting ES cell self renewal. Large differentiated cells typically found at the borders of ES cell colonies had increased levels of K8, K18 (Figure 4E) and K19 (Figure 4A). Human ES cells, unlike mES cells, spontaneously differentiate to trophoblast lineage cells. K7 has been found to be a useful marker of human trophoblast lineage [21]. While K7 positive cells are relatively rare in hES cultures they can be detected as discrete colonies of presumptive trophoblast derivatives within undifferentiated hES cell neighbors in cultures incubated for a week or longer. In freshly passaged cells K7 positive cells were detected as dispersed single cells, apparently due to the dissociation of rare colonies of presumptive trophoblasts. (Figure 5A, B). Supporting evidence that the K7 expression reflects trophoblast formation is provided by the correlation of K7 RNA with the CDX2 homeobox master regulatory transcription factor (Figure 5C) in a data set that includes both undifferentiated and differentiated hES cells [17]. Furthermore the expression of CDX2 RNA was inversely correlated with OCT4 RNA (Figure 5D), as expected from the known repression of CDX2 by OCT4 [22], [23].

**Figure 6:**
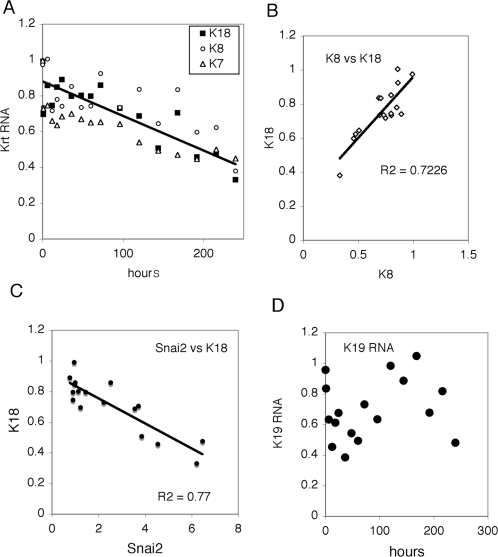
Decreased keratin expression during neuronal differentiation. Averages o fat least two samples for each time point were used to measure K7, K8, K18 and K19 RNAs as a function of hours after starting induction of neuronal progenitor differentiation. A, K7, K8 and K18 RNA levels over time course of 240 hours. Trendline is for K18 data. B, correlation of K8 and K18 RNA levels during neuronal progenitor differentiation. C, negative correlation of K18 RNA and Snai2 RNA levels. D, K19 RNA levels do not reveal a simple trend.

Simple epithelia keratins are expressed only in the ependymal layer of the ventricles of the adult brain. The differentiation of hES to neural tissues can now be performed routinely. We have confirmed that during the accelerated differentiation of hES cells to neuronal progenitor cells expression of K8 and K18 are decreased (Figure 6A). The K8 and K18 RNAs appear coordinately suppressed (Figure 6B). However, this suppression is not due to decreased Jun RNA expression as there is no correlation of K18 and Jun RNAs (data not shown). Repression is correlated with increased expression of Snai2 (Slug) a transcriptional repressor associated with neural crest formation and epithelial-mesenchymal transition (Figure 6C). Snai2 has previously been suggested as active in K8 repression [24]. Unlike the RNAs for K8 and K18, K19 RNA levels do not reveal a simple trend upon the induction of neuronal progenitor differentiation (Figure 6D). Thus the mechanisms for suppression of K8 and K18 RNAs do not appear to extend to K19 regulation.

## Discussion

Some investigations of the hES cells have reported unexpected expression of K8 and K18 because these gene products were expected to be differentiation markers. [9]. Some of these early studies suggested that expression might be due to contamination from differentiated cells or possibly translational regulation. However, a meta analysis of the results of over 38 studies of gene expression in hES cells confirms that K8 and K18 RNAs are commonly found in undifferentiated hES cells [19] and are generally increased in differentiated cells from embryoid bodies. We have confirmed that undifferentiated hES cells express simple epithelial keratin RNAs and proteins as filament networks, typical of other simple epithelial cells, and by contrast with mouse ES cells.

The differences in K8 and K18 expression in mouse and hES cells may reflect a fundamental difference between the embryonic equivalent of the mouse and human inner cell masses. The human epiblast is an epithelial structure while the mouse ICM does not adopt an epithelial organization until after blastocyst implantation. Keratin expression in hES cell lines reflects the more flattened epithelial phenotype of hES colonies compared to mouse ES cells. The expression of keratins may reflect differences in the originating cell types of the respective blastocysts or differences in the the state of acquisition of a stable, self-replicative capacity. The suppression of K8 and K18 expression in the mouse inner cell mass is an active process that may correspond to the transcriptional inhibitory activity detected in embryonal carcinoma cells [13]. The increased expression of K8 and K18 in mouse epiblast stem cells (EpiSC) is consistent with the suggestion that both ES and EpiSC cells lines reflect the characteristics of the embryonic tissue of origin. In transplantation experiments human specific K8, K18 or K19 antibodies may aid in identifying both hES cells and their differentiated progeny.

Mouse EpiSC are poorly compatible with embryo chimerism, at least by standard ES cell methods of blastocyst injection and morula aggregation, but retain the ability to differentiate to multiple tissues as judged by teratoma formation and in vitro differentiation [10], [11]. The strong intercellular adhesive and epithelial nature of EpiSC and hES cells may challenge the integration of EpiSC into preimplantation embryos. Thus ES cells are preferable for gene knockout studies. Mouse EpiSC do provide the opportunity of investigating maintenance of the pluripotent state and perhaps model hES cells. Speculative extrapolation of the similarity between hES cells and mouse EpiSC might question the compatibility of hES cells with early embryonic chimerism.

Differences in expression of simple epithelial keratins in mouse and human ES cells may also have direct consequences on hES cells. These keratins have been implicated in resistance against death receptor mediated apoptosis [25], [26], [27] and stress, possibly through the titration of phospho-protein signaling molecules [26], [28]. Phosphorylated K8/18 networks can titrate phospho-protein binding proteins such as 14-3-3 isoforms and thus impact cell proliferation [29]. Furthermore, expression of the relatively insoluble subunits of simple epithelial keratins carries the risk of protein aggregation induced cellular disease [30]in the event of mutation, imbalance of subunit expression, chemical induced aggregation or deficient degradation [31].

While expression of keratins in hES cells is substantial, accumulated expression in some cells can be much higher. For example in the MCF7 human breast cancer cell line, K18 is among the most abundant proteins within the cells. Similarly, spontaneous differentiated cells arising in hES cultures contain substantially higher accumulation of keratin proteins. The stability and abundance of individual keratins makes them excellent cell type or lineage markers. However, the molecular mechanisms responsible for the cell type specific expression are still obscure. The very close correspondence of RNA levels of K8 and K18 very likely reflect their coordinate regulation from adjacent locations at the distal end of the type II keratin locus on chromosome 12. Both genes are regulated by AP-1 and Ets transcription factor families [14], [32]. Jun activates the K18 gene from an enhancer located in the first intron and from a regulatory element embedded within a coding exon [33]. The coordinate regulation of K8 and K18 may reflect the recent identification of CTCF insulator protein binding sites flanking the two genes on chromosome 12 [34] that suggests a chromosomal regulatory domain. In contrast, the coordinate expression of the K8 and K19 genes occurs despite the separate chromosomal locations of K8 on chromosome 12 and K19 on chromosome 17. The basis of coordinate regulation of pairs of type I and II keratins is not known.

## Materials and Methods

### RNA expression analysis

Primary RNA expression data from mouse ES cells [35] (Agilent platform, GSE3231), human ES cells [17] (Illumina platform), (www.stemcellcommunity.org) and mouse EpiSC and ES cells (Agilent platform, GSE7902) [10] were downloaded and examined for keratin gene expression in Excel. For some comparisons of different cell types, keratin expression was normalized to signals of same arrays for beta-actin although this normalization did not change the results greatly. Confirmation of hES cell expression of keratins was confirmed in array results available through Amazonia! public database [19] (http://amazonia.montp.inserm.fr/).

Differentiating human ES cells RNAs were isolated with the use of Trizol reagent (Sigma Chemical, St. Louis, Mo). Labeled cRNA was prepared from 500 ng RNA using the Illumina® RNA Amplification Kit from Ambion (Austin, TX, USA). The Biotin labeled cRNA (750 ng) was hybridized 18 hr at 58°C to the HumanRef-8 v2 Expression BeadChip. (Illumina, San Diego, CA, USA) according to the manufacturer's instructions. BeadChips were scanned with an Illumina BeadArray Reader and hybridization efficiency was monitored using BeadStudio software (Illumina) and internal controls built into the Illumina system. Gene expression data was imported into Genespring software.

### Cell culture

Human ES cell lines H9 and H14, were cultivated on inactivated mouse fibroblast feeder layers or on growth factor reduced Matrigel coated plastic in Knockout DMEM (Invitrogen) supplemented with 200 mM glutamine, 0.1 mM 2-mercaptoethanol [36] 20% Knockout serum substitute (Invitrogen) and 25 ng/ml recombinant human basic FGF (Sigma, St. Louis, MO) as described [37], [38]. Hues7 and Hues13 cells were propagated as described [39]. D9 mouse ES cells, HR9 mouse extraembryonic endodermal cells and mouse trophoblast stem cells were cultivated as previously described [3], [40], [41]. MCF7 and CaCo2 human tumor cells were obtained from ATCC.

Methods and characterization of accelerated hES cell differentiation to neural progenitors, will be described in detail elsewhere (Bajai, R and Terskikh, A, submitted). In short, small clusters (10–100 cells) of hESCs were grown in uncoated dishes (Costar) in 1∶1 ratio of DMEM/ and F12 medium with N2 (Gibco) and B27 (Gibco) factor supplements, 20 ng/ml insulin 20 ng/ml bFGF, 20 ng/ml EGF and 2 mM N-acetyl cysteine (NAC)). The spheres were grown for 6–8 days, with a change in medium every alternate day. Spheres were collected, gently triturated and plated on ornithine coated (5 ng/ml, Sigma) plates in DMEM/F12, 10% BIT 9500 supplement (Stem Cell Technologies, ), 20 ng/ml bFGF, 20 ng/ml EGF, 5 ug/ml fibronectin, 2 ug/ml heparin).

### Protein analysis

For western blot analysis of total keratin proteins, 6 cm dishes of PBS washed cells were dissolved in 200 ul of 9.5 M urea, heated to 100c for 5 min, mixed vigorously and cleared of minimal residual debris by centrifugation. Samples were dilute with concentrated SDS sample buffer and separated in low bis acrylamide gels as previously described [3]. Proteins were blotted onto PVDF membranes according to the instructions of the supplier of the secondary antibodies (Li-COR Bioscience) and detected with 680 or 800 CW IR-dye labeled secondary antibodies. Reaction was imaged and quantitated in a LI-COR Odyssey image analyzer. Filters were stripped in 62.5 mM TrisHCL, pH 6.8, 2% SDS, 100 mM 2-mercaptoethanol at 55C for 1 hour and re-probed with antibody to beta-tubulin (E7 mouse monoclonal antibody, (Developmental Studies Hydridoma Bank, Iowa City, Iowa). Results are shown for antibodies that detected both human and mouse form of rat monoclonal antibody to K8 (TROMA1) [6] (gift from Rolf Kemler, available from Developmental Studies Hydridoma Bank); rabbit polyclonal antiserum to mouse K18 (1589) [42] and mouse monoclonal antibody to K19 (L2PK) [43], [44] (gift from Dr. Birgit Lane) are shown. Results were also confirmed with the following antibodies that were species dependent: mouse monoclonal to K8, M20 (Sigma); rat monoclonal to mouse K18, TROMA2 [6], mouse monoclonal to human K18, CK5 (Sigma); mouse monoclonal to human K19, K4.62 (Sigma).

### Immunofluorescence

Cells were plated on feeder layer or Matrigel coated glass coverslips, fixed in cold methanol for 10 minutes, rinsed and incubated with PBS-T (PBS^Ca+/Mg+^+0.1% Tween20) for one hour at room temperature. Cells were blocked for 20 minutes in PBS supplemented with 2% goat serum (Gibco). For combined detection of Oct3/4 and K19, Oct3/4 antibody (H-134, sc-9081 rabbit polyclonal from Santa Cruz) was diluted 1∶250 in 1% goat serum (GS) in PBS and incubated with the cells overnight at 4°C. Cells were washed with PBS-T and PBS. The second primary antibody (K19, LP2K monoclonal cell culture supernatant) was used neat for one hour at room temperature. After washing, secondary antibodies against the species were combined and diluted 1∶500 in 1% GS-PBS (Alexa 488 anti-mouse and Alexa 546 anti-rabbit from Invitrogen). Cells were incubated with the secondary antibodies for one hour at room temperature in the dark. Washes were repeated as described above. Finally cells were incubated with a DAPI solution for 3 minutes followed by two PBS washes. Other keratins were detected by incubating with either TROMA1 (rat antiK8), M20 (mouse antiK8, 1∶200), 1589 (rabbit polyclonal antiK18, 1∶40), RCK105 (mouse monoclonal antiK7, 1∶2, gift from F. Ramaekers) and the appropriate secondary antibody and the same blocking and washing procedures. Images were acquired on an inverted TE300 Nikon or an OlympusIX71 fluorescence microscope with a Diagnostic Instrument cooled color CCD SPOT RT camera or Hamamatsu Digital Camera respectively. Images were combined in either SPOT or MetaVue 7.1.6.0 software and were formatted for publication with Photoshop software.
